# Leukocyte cell-derived chemotaxin 2 inhibits development of atherosclerosis in mice

**DOI:** 10.24272/j.issn.2095-8137.2019.030

**Published:** 2019-07-18

**Authors:** Wen-Ming He, Ting Dai, Jiong Chen, Jian-An Wang

**Affiliations:** 1 Department of Cardiology of the Second Affiliated Hospital, Zhejiang University School of Medicine, Hangzhou Zhejiang 310009, China; 2 Department of Cardiology, the Affiliated Hospital of Medical School of Ningbo University, Ningbo Zhejiang 315010, China; 3 Laboratory of Biochemistry and Molecular Biology, School of Marine Sciences, Meishan Campus, Ningbo University, Ningbo Zhejiang 315832, China

**Keywords:** Leukocyte cell-derived chemotaxin 2 (LECT2), Atherosclerosis, Inflammation, Lipid metabolism

## Abstract

Leukocyte cell-derived chemotaxin 2 (LECT2), a multifunctional hepatokine, is involved in many pathological conditions. However, its role in atherosclerosis remains undefined. In this study, we administered vehicle or LECT2 to male *Apoe^-/-^* mice fed a Western diet for 15 weeks. Atherosclerotic lesions were visualized and quantified with Oil-red O and hematoxylin staining. The mRNA expression levels of MCP-1, MMP-1, IL-8, IL-1β, and TNF-α were analyzed by quantitative real-time polymerase chain reaction. Serum TNF-α, IL-1β, IL-8, MCP-1, and MMP-1 concentrations were measured by enzyme-linked immunosorbent assay. CD68, CD31, and α-SMA, markers of macrophages, endothelial cells, and smooth muscle cells, respectively, were detected by immunostaining. Results showed that LECT2 reduced total cholesterol and low-density lipoprotein concentrations in serum and inhibited the development of atherosclerotic lesions, accompanied by reductions in inflammatory cytokines and lower MCP-1, MMP-1, TNF-α, IL-8, and IL-1β mRNA abundance. Furthermore, LECT2 decreased CD68, but increased α-SMA in atherosclerotic lesions, suggesting an increase in smooth muscle cells and reduction in macrophages. In summary, LECT2 inhibited the development of atherosclerosis in mice, accompanied by reduced serum total cholesterol concentration and lower inflammatory responses.

## INTRODUCTION

Cardiovascular diseases are the leading cause of mortality worldwide. With ageing populations and increasing exposure to metabolic risks, deaths due to cardiovascular diseases will increase to approximately 23 million by 2030 (GBD 2017 Risk Factor Collaborators, 2018). Atherosclerosis is an important underlying cause of many clinical cardiovascular events, and is a systemic disease characterized by fatty deposits, inﬂammation, cell death, fibrosis, and scar tissue build up within the walls of arteries (Mozaffarian et al., 2016). In the early stages of atherosclerosis, a large amount of lipids and proinflammatory mediators accumulate in macrophages within the arterial wall, which become foam cells (Lim & Park, 2014). As the disease advances, vascular smooth muscle cells (VSMCs) migrate from the media to intima, and then proliferate and produce extracellular matrix to form a fibrous cap that covers atherosclerotic plaque (Allahverdian et al., 2018). During this stage, the secretion of many proinflammatory cytokines is increased, including interleukin (IL)-8, IL-1β, monocyte chemoattractant protein-1 (MCP-1), and tumor necrosis factor (TNF)-α (Chistiakov et al., 2015). In the advanced stage, macrophages induce VSMC apoptosis via the TNF-α/NO signaling pathway (Boyle et al., 2003) and reduce collagen synthesis by secretion of matrix metalloproteinases (MMP) (Müller et al., 2014). Although the precise pathogenesis of atherosclerosis has not been clarified, it is now widely accepted that inflammation and lipid disorder both play crucial roles (Ference et al., 2017; Hansson & Libby, 2006).

Recent studies have demonstrated that a set of predominantly liver-derived proteins can directly affect the progression of atherosclerosis (Yoo & Choi, 2015). Leukocyte cell-derived chemotaxin 2 (LECT2) is a 16 kDa secretory protein first isolated from cultured supernatants of phytohemagglutinin-activated human T-cell leukemia SKW-3 cells. It is predominantly secreted by hepatocytes (Yamagoe et al., 1998) and is generally expressed in vascular cells, endothelial cells, and VSMCs (Slowik & Apte, 2017). Previous studies have reported that LECT2 has an inhibitory effect on inflammation. In mice, LECT2 deficiency can cause severe arthritis, with reductions in the production of cytokines and chemokines, e.g., IL-1β, IL-6, TNF-α, and MCP-1, after exogenous LECT2 injection (Okumura et al., 2008). We recently reported that LECT2 can protect mice against bacterial sepsis by enhancing phagocytosis and bacterial killing of macrophages via CD209a (Lu et al., 2013) and can also induce hematopoietic stem cell expansion and mobilization (Lu et al., 2016). In human hepatocellular carcinoma, low LECT2 expression is correlated with advanced histological grade and inflammatory infiltrates. L'Hermitte et al. (2019) showed that LECT2-deficient hepatocellular carcinoma cells can secrete chemotactic signals, resulting in amplification of inflammatory monocytes, which harbor an immature phenotype with immunosuppressive capacities and tumor-promoting potential. In addition, Zhang et al. (2018) discovered that circulating LECT2 concentrations are significantly higher in newly diagnosed type-2 diabetic patients, especially those that are obese, and that concentrations of LECT2 are negatively associated with high-density lipoprotein-cholesterol levels. Researchers have also found that LECT2 is involved in many other pathological conditions, such as obesity (Sargeant et al., 2018), skeletal muscle insulin resistance (Jung et al., 2018), non-alcoholic fatty liver disease, and metabolic syndrome (Yoo et al., 2017), thus suggesting the potential role of LECT2 in atherosclerosis. However, such roles remain undefined. The purpose of this study was to investigate the effects of LECT2 on atherosclerosis in *Apoe^-/-^* mice.

## MATERIALS AND METHODS

### Animals

Male *Apoe^–/–^* and C57BL/6 wild-type mice (6 weeks old, 20±2 g) were purchased from Beijing Vital River Laboratory Animal Technology (China). The C57BL/6 mice were fed a normal laboratory diet and used as blank controls, whereas the *Apoe^–/–^* mice were fed a Western diet (0.15% w/w cholesterol, 40 kcal% butter fat, Beijing Biotech-HD) for 15 weeks as an atherosclerosis model. All mice were housed in a constant temperature (21±2 °C) room, under a 12 h dark/12 h light cycle in a pathogen-free environment in the Animal Care Facility of Ningbo University Medical School according to the institutional guidelines.

### *In vivo* application of LECT2

Mice were randomly divided into three groups: i.e., (1) control group (C57BL/6 mice, PBS 100 µL, subcutaneous (sc), *n*=8), (2) atherosclerosis (AS) group (*Apoe^–/–^* mice, PBS 100 µL, sc, *n*=8), and (3) LECT2 group (*Apoe^–/–^* mice, LECT2 0.2 mg/kg, sc, *n*=8). Each mouse received a sc injection every 2 d from 6 to 21 weeks of age. LECT2 was dissolved in PBS and kept at 4 °C for 2 d. Two days after the last injection, mice were euthanized to analyze and characterize atherosclerosis.

### Recombinant LECT2 protein

Recombinant mouse LECT2 proteins (purity: 96.20%) were produced from CHO cells, as described in our previous research (Lu et al., 2013, 2016).

### Measurement of lipid parameters

Two days after final administration, all mice were sacrificed after retro-orbital bleeding. All blood samples were centrifuged at 3 000 r/min for 15 min at room temperature to collect serum. Commercially available kits were then used to measure total cholesterol (TC), total triglyceride (TG), high-density lipoprotein cholesterol (HDL-c), and low-density lipoprotein cholesterol (LDL-c) concentrations according to the manufacturer’s protocols.

### LECT2 detection

The ELISA system used one antibody as the capture antibody (rabbit anti-LECT2, C-terminal; Santa Cruz, CA, USA.) and another for detection (goat anti-LECT2, N-terminal; Santa Cruz, CA, USA.).

### ELISA for cytokines

Serum concentrations of inflammatory cytokines, TNF-α (MTA00B, R&D Systems , Minneapolis, MN, USA ), IL-1β (MLB00C, R&D Systems , Minneapolis, MN, USA), IL-8 (SBJ-M0010, Senbeijia Bio. Co, Nanjing, China), MMP-1 (CSB-E07417m, CUSABIO, China), and MCP-1 (CSB-E07430m, CUSABIO, China) were measured with commercial ELISA kits according to the manufacturer’s instructions. Absorbance was measured using a Multiskan Ascent plate reader (Thermo Electron Corporation, Waltham, Massachusetts, USA ).

### Histological evaluation

After termination, the periaortic tissue around the aorta was cleaned, and the whole aorta from the heart to the abdominal aorta was dissected. Atherosclerotic plaque in the aortas was stained with 0.5% Oil-red O (Sigma-Aldrich) for 15 min at room temperature. After staining, the aortas were washed with 70% ethanol and then distilled water. Subsequently, the whole aorta was cut open and flattened on a black plate to take pictures. Aortic roots were embedded in optimal cutting temperature compound (Sakura, Torrance, CA, USA) and frozen in ultra-cold isopentane on dry ice. Serial cryosections (8 μm) were cut along the aortic root specimens at –20 °C using a cryotome (HM550, Thermo Scientific, Rockford, IL, USA). At least three transverse sections (spaced around 60 μm) from each aortic root were stained with hematoxylin/eosin for histology analyses. Oil-red O staining was performed to evaluate the lipid content in the plaque area. Image Pro-Plus 6.0 (Media Cybernetics, USA) was used to analyze aorta images, following the recent AHA Statement (Daugherty et al., 2017).

### Immunohistochemistry (IHC)

IHC staining was performed on 8 μm frozen sections of the aortic root to detect the proportion of macrophages, endothelial cells, and SMCs. Frozen sections were air-dried, fixed with cold acetone, and incubated in anti-CD31 antibody (1:50 dilution; ab28364, Abcam, USA), anti-CD68 antibody (1:50 dilution; ab125212, Abcam, USA), and anti-alpha smooth muscle actin antibody (1:200 dilution; ab5694, Abcam, USA), respectively, at 4 °C overnight. Substitution of PBS for the specific primary antibody was performed as a negative control. All steps were operated following the instruction manual of the SABC (rabbit IgG)-POD kit (Solarbio, Beijing, China). Finally, slides were stained with DAB, counterstained with hematoxylin, dehydrated, and mounted. Images were detected using a Nikon A1R confocal laser scanning microscope (Nikon, Tokyo, Japan). Image Pro-Plus 6.0 software (Media Cybernetics, Rockville, USA) was used to analyze the positively stained areas. At least three sections were analyzed per aortic root.

### Total RNA isolation and quantitative real-time polymerase chain reaction (qRT-PCR)

Aorta total RNA (five mice from control, AS, and LECT2 groups) was extracted by Trizol reagent (Invitrogen, Carlsbad, CA, USA) following the manufacturer’s protocols. Complementary DNA (cDNA) was synthesized using a HiFiScript first-strand cDNA synthesis kit (ComWin Biotech, Beijing, China) according to the manufacturer’s instructions. PCR amplification was accomplished using 1×FastStart Essential DNA Green Master (Roche, Mannheim, Germany) with a LightCycler 480 II instrument (Roche, Switzerland). The primer sequences used included (forward and reverse): MCP-1, 5'-TTAAAAACCTGGATCGGAACCAA-3' and 5'-GCATTAG CTTCAGATTTACGGGT-3'; MMP-1, 5'-TGTTTGCAGAGCAC TACTTGAA-3' and 5'-CAGTCACCTCTAAGCCAAAGAAA-3'; IL-8, 5'-TCGAGACCATTTACTGCAACAG-3' and 5'-CATTGCC GGTGGAAATTCCTT-3'; IL-1β, 5'-AGAAGCTGTGGCAGCTA-3' and 5'-TGAGGTGCTGATGTACCA-3'; TNF-α, 5'-GAACTGG CAGAAGAGGCACT-3' and 5'-GGTCTGGGCCATAGAACTG A-3'; and 18S rRNA, 5'-TTTGTTGGTTTTCGGAACTGA-3' and 5'-CGTTTATGGTCGGAACTACGA-3'. Data were normalized to 18S rRNA and the dosage of the target fragments was calculated using the 2^–ΔΔCT^ method. Sequences were confirmed using NCBI BLAST software.

### Statistical analysis

Results were presented as means±standard deviation (*SD*). Statistical analysis was performed using GraphPad Prism 7.0 (GraphPad Software, San Diego, CA, USA). The Shapiro-Wilk test was used to check the normality of the data. If data displayed normal distribution, an unpaired Student’s *t*-test was used to compare two groups. If the data did not display normal distribution, the Mann-Whitney U test was performed.

## RESULTS

### **LECT2 inhibited development of atherosclerotic lesions in *Apoe*** *^–^* ***^/^*** *^–^* **mice**

**Figure 1:**
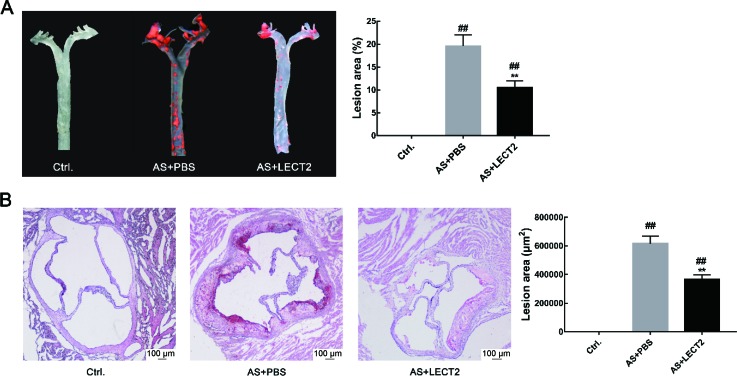
**LECT2 attenuated development of atherosclerotic lesions in *Apoe*** *^–^* ***^/^*** *^–^* **mice** A: Representative en-face images of Oil-red O-stained aortas from control, AS, and LECT2 groups (*n*=3 per group). The percentage of atherosclerotic plaque area to total aortic surface area was measured using an en-face method. B: Oil-red O staining of aortic sinus sections (*n*=8 per group). Data are means±*SD*. ##: *P*<0.01, vs. control; **: *P*<0.01, vs. AS group. Scale bars: 100 μm.

We analyzed the levels of LECT2 in the AS and control groups to examine the relationship between serum LECT2 levels and atherosclerosis. Results showed that the LECT2 levels in the AS group (33.37±2.85 ng/mL, *n*=6) were significantly lower than that in the control group (43.62±2.20 ng/mL, *n*=6; *P*<0.05). Oil-red O staining showed no obvious atherosclerotic plaque in the control group aortas, but apparent plaque in the aortas of the AS group. The LECT2 group had less atherosclerotic plaque in the ascending aortic arch and thoracic regions compared with the AS group (*P*<0.001) (Figure 1A). Consistently, hematoxylin and Oil-red O staining of the aortic sinus for atherosclerotic lesions also showed reduced lipid accumulation in the LECT2 group compared with the AS group (*P*<0.001) (Figure 1B).

### **LECT2 reduced serum lipids in *Apoe*** *^–^* ***^/^*** *^–^* **mice**

**Figure 2:**
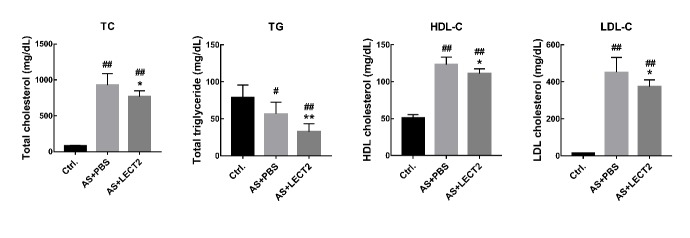
Effects of LECT2 on lipid profile in serum *Apoe^–/–^* mice were fed a Western diet from 6 weeks of age and were treated with 0.2 mg/kg LECT2 every 2 d. A–C: Plasma total cholesterol (TC), triglyceride (TG), and low-density lipoprotein cholesterol (LDL-c) concentrations at 21 weeks of age (*n*=8 per group). Data are means±*SD*. #: *P*<0.05, ##: *P*<0.01 vs. control; *: *P*<0.05, **: *P*<0.01, vs. AS group.

There is a close connection between dyslipidemia and atherosclerosis (Wang et al., 2018). Compared with the AS group, LCET2 administration reduced the serum total cholesterol (927±160 mg/dL, 768±79 mg/dL, *P*<0.05), TG (57±16 mg/dL, 33±11 mg/dL, *P*<0.01), HDL-c (123±10 mg/dL, 111±7 mg/dL, *P*<0.05), and LDL-c concentrations (452±81 mg/dL, 374±37 mg/dL, *P*<0.05) (Figure 2).

### LECT2 reduced mRNA abundance of inflammatory cytokines and chemokines

**Figure 3:**
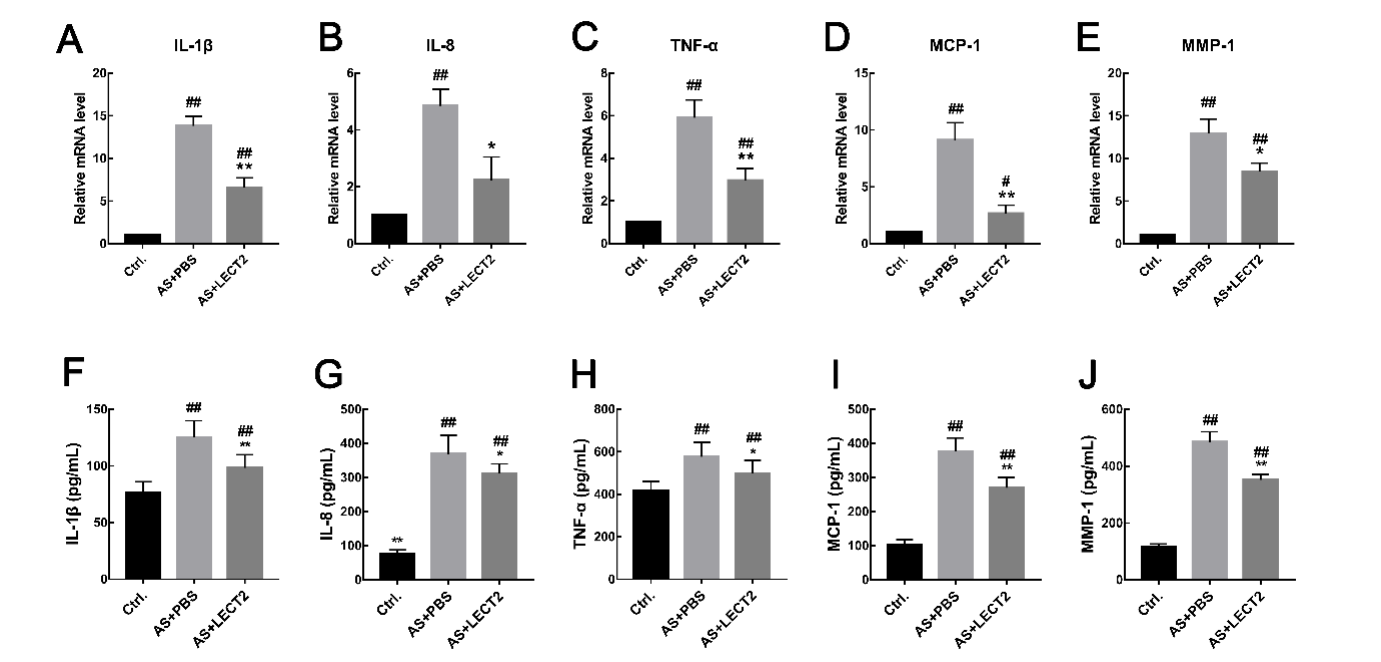
**LECT2 reduced mRNA expression and serum concentrations of inflammatory cytokines and chemokines in *Apoe*** *^–^* ***^/^*** *^–^* **mice** mRNA expression of IL-1β (A), IL-8(B), TNF-α(C), MCP-1(D), and MMP-1(E) was quantified by qRT-PCR (*n*=5 per group). And the serum concentrations were determined using enzyme-linked immunosorbent assay (*n*=8 per group). Data are means±*SD*. #: *P*<0.05, ##: *P*<0.01, vs. control; *: *P*<0.05, **: *P*<0.01, vs. AS group.

We performed qRT-PCR to confirm the effect of LECT2 on MCP-1, MMP-1, TNF-α, IL-1β, and IL-8 mRNA expression. Consistent with the systemic inflammation findings, the mRNA expression levels of these inflammatory cytokines and chemokines in the whole aorta were lower in the LECT2 group than that in the AS group (Figure 3A–E). Furthermore, results showed a dose-dependent relationship, whereby the inflammatory cytokines and chemokines decreased with the increase in LECT2 concentration (data not shown).

### **LECT2 led to lower serum inflammatory cytokine concentrations in *Apoe*** *^–^* ***^/^*** *^–^* **mice**

Inflammation is a critical biological process in atherosclerosis (Wolf & Ley, 2019). Compared with the control group, serum concentrations of inflammatory cytokines and chemokines, including TNF-α (*P*<0.05), IL-1β (*P*<0.01), IL-8 (*P*<0.01), MCP-1 (*P*<0.001), and MMP-1 (*P*<0.001), were higher in the AS group. Administration of LCET2 led to reductions of these inflammatory cytokines (Figure 3F–J).

### LECT2 changed atherosclerotic plaque composition

**Figure 4:**
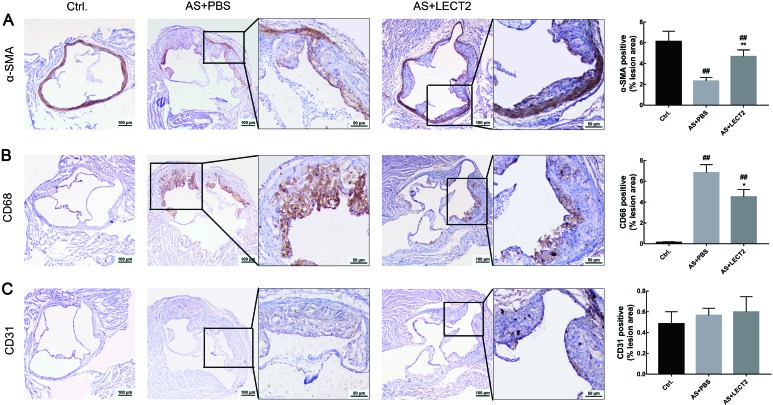
LECT2 changed atherosclerotic plaque composition Sections of aortic root from control, AS, and LECT2 groups (*n*=8 per group) immunostained for smooth muscle cells (A: α-SMA), macrophages (B: CD68), and endothelial cells (C: CD31). Positive signals are shown in brown. Sections were counterstained with hematoxylin. Data are means±*SD*. ##: *P*<0.01, vs. control; **: *P*<0.01, vs. AS group. Scale bars: 100 μm.

We assessed the proportion of smooth muscle cells, macrophages, and endothelial cells by IHC staining to determine whether LECT2 affected the composition of atherosclerotic plaque. Compared with the AS group, LECT2 administration increased the number of smooth muscle cells (Figure 4A) and reduced the proportion of CD68 macrophages (Figure 4B) but had no influence on the proportion of CD31 endothelial cells in the lesions (Figure 4C).

## DISCUSSION

In the present study, we investigated the effects of LECT2 on the development of atherosclerosis in mice. Results demonstrated that LECT2 exhibited an anti-atherosclerotic function, accompanied by a reduction in serum total cholesterol concentration and lower inflammatory responses.

We first measured atherosclerotic lesions in the aortas of *Apoe* ***^–/–^*** mice and found that LECT2 inhibited atherosclerosis, resulting in a 46% reduction in total plaque size. In addition to the observed reduction in plaque, LECT2 also had an effect on the serum lipid profile. The liver plays a central role in regulating whole body cholesterol homeostasis. Hepatocytes maintain cellular cholesterol homeostasis by controlling several cholesterol input and elimination pathways. These input pathways are primarily regulated by the sterol regulatory element-binding protein (SREBP)-2-mediated cholesterol sensing mechanism (Ye & DeBose-Boyd, 2011). In earlier studies, Hwang et al. (2015) showed that LECT2 increases mTOR phosphorylation and SREBP-1 cleavage in hepatocytes, leading to hepatic lipid accumulation; however, the study did not show serum lipid levels or SREBP-2 expression. Mammalian cells produce three SREBP isoforms, which control the anabolic pathways of cholesterol, free fat acids, and triglycerides (Engelking et al., 2018). SREBP-1a and SREBP-1c are active in driving the transcription of genes involved in fatty acid synthesis, whereas SREBP-2 is more active in stimulating transcription of genes involved in cholesterol biosynthesis (Ye & DeBose-Boyd, 2011). Based on our results, we hypothesize that LECT2 may reduce serum TG, TC, and LDL-c concentrations and contribute to its anti-atherosclerotic effects by impacting the SREBP pathways. However, this needs to be corroborated by subsequent studies.

In addition to lipid accumulation, the inflammatory mechanism is another important factor in the pathogenesis and clinical manifestation of atherosclerosis. In the process of atherosclerosis, proinflammatory factors, including IL-1β and IL-8, are expressed by ox-LDL-stimulated endothelial cells and VSMCs, which, in turn, activate macrophages, resulting in amplification of the inflammatory response (Chistiakov et al., 2015; Wolf & Ley, 2019). The MCP-1 chemokine, also known as CC-chemokine ligand 2, plays an important role in the recruitment of monocytes to atherosclerotic lesions, and also contributes to thrombin generation and thrombus formation by generating tissue factor (Charo & Taubman, 2004). Han et al. (1998) reported that elevated plasma LDL concentrations can enhance C-C chemokine receptor type 2 (CCR2, receptor for MCP-1) expression and chemotactic response. Thus, LECT2 may reduce MCP-1 expression by decreasing LDL-c concentrations. On the other hand, inflammation can increase cellular cholesterol uptake and synthesis via up-regulation of low-density lipoprotein receptor and 3-hydroxy-3-methylglutaryl-coenzyme A reductase, but can decrease cholesterol efflux via down-regulation of liver X receptor alpha and ATP-binding cassette transporter A1 (Zhong et al., 2015), implying that inflammation promotes lipid accumulation and foam cell formation by disrupting cellular cholesterol homeostasis. Previous research has also shown that LECT2 is a direct target gene of the Wnt/β-catenin signaling pathway, and β-catenin negatively regulates nuclear transcription factor (NF-κB) signaling, which plays an important role in the regulation of inflammation responses (Ovejero et al., 2004). The results of our study are consistent with the above theory. We proved that circulating LECT2 levels were significantly lower in atherosclerotic mice, suggesting that LECT2 has an inhibitory effect on atherosclerosis. LECT2 mitigated the expression and secretion of IL-1β, IL-8, TNF-α, and IL-1β (Figure 3) dose-dependently in *Apoe* ***^–/–^*** mice fed with a Western diet, suggesting that LECT2 may inhibit atherosclerosis by alleviating inflammatory responses via Wnt/β-catenin signaling. In addition, macrophage-derived MMPs, which play a potential role in fibrous cap thinning, are thought to promote vascular smooth muscle cell growth and extracellular matrix and atherosclerotic plaque homeostasis (Johnson, 2007) via degradation of various extracellular matrix proteins, including collagens (Moore & Tabas, 2011). We found that LECT2 also increased the content of SMCs but reduced the proportion of macrophages (Figure 4), likely reflecting the role of LECT2 in regulating the composition of atherosclerotic plaque.

In conclusion, we showed, for the first time, that LECT2 inhibits the development of atherosclerosis in a hypercholesterolemic mouse model, accompanied by reduced serum total cholesterol concentration and lower inflammatory responses. Our future studies will aim to define the mechanisms involved in LECT2 prevention of atherosclerosis development.

## References

[ZoolRes-40-4-317-R1] Allahverdian S, Chaabane C, Boukais K, Francis GA, Bochaton-Piallat ML 2018 Smooth muscle cell fate and plasticity in atherosclerosis. Cardiovascular Research, 114(4): 540–550. 29385543 10.1093/cvr/cvy022 PMC5852505

[ZoolRes-40-4-317-R2] Boyle JJ, Weissberg PL, Bennett MR 2003 Tumor necrosis factor-alpha promotes macrophage-induced vascular smooth muscle cell apoptosis by direct and autocrine mechanisms. Arteriosclerosis, Thrombosis, and Vascular Biology, 23(9): 1553–1558. 10.1161/01.ATV.0000086961.44581.B7 12869351

[ZoolRes-40-4-317-R3] Charo IF, Taubman MB 2004 Chemokines in the pathogenesis of vascular disease. Circulation Research, 95(9): 858–866. 15514167 10.1161/01.RES.0000146672.10582.17

[ZoolRes-40-4-317-R4] Chistiakov DA, Orekhov AN, Bobryshev YV 2015 Vascular smooth muscle cell in atherosclerosis. Acta Physiologica, 214(1): 33–50. 25677529 10.1111/apha.12466

[ZoolRes-40-4-317-R5] Daugherty A, Tall AR, MJAP Daemen , Falk E, Fisher EA, Garcia-Cardena G, Lusis AJ, Owens P, III, Rosenfeld ME, Virmani R 2017 Recommendation on design, execution, and reporting of animal atherosclerosis studies: A scientific statement from the american heart association. Arteriosclerosis, Thrombosis, And Vascular Biology, 37(9): E131–E157. 10.1161/ATV.0000000000000062 28729366

[ZoolRes-40-4-317-R6] Engelking LJ, Cantoria MJ, Xu Y, Liang G 2018 Developmental and extrahepatic physiological functions of SREBP pathway genes in mice. Seminars in Cell & Developmental Biology, 81: 98–109. 28736205 10.1016/j.semcdb.2017.07.011 PMC5775927

[ZoolRes-40-4-317-R7] Ference BA, Ginsberg HN, Graham I, Ray KK, Packard CJ, Bruckert E, Hegele RA, Krauss RM, Raal FJ, Schunkert H, Watts GF, Borén J, Fazio S, Horton JD, Masana L, Nicholls SJ, Nordestgaard BG, van de Sluis B, Taskinen MR, Tokgözoğlu L, Landmesser U, Laufs U, Wiklund O, Stock JK, Chapman MJ, Catapano AL 2017 Low-density lipoproteins cause atherosclerotic cardiovascular disease. 10.1093/eurheartj/ehx144 PMC5837225 28444290

[ZoolRes-40-4-317-R8] Evidence from genetic, epidemiologic, and studies Oclinical . A consensus statement from the European Atherosclerosis Society Consensus Panel. European Heart Journal, 38(32): 2459–2472. 10.1093/eurheartj/ehx144 PMC5837225 28444290

[ZoolRes-40-4-317-R9] GBD 2017 Risk Factor Collaborators 2018 Global, regional, and national comparative risk assessment of 84 behavioural, environmental and occupational, and metabolic risks or clusters of risks for 195 countries and territories, 1990-2017: a systematic analysis for the Global Burden of Disease Study 2017. Lancet, 392(10159): 1923–1994. 30496105 10.1016/S0140-6736(18)32225-6 PMC6227755

[ZoolRes-40-4-317-R10] Han KH, Tangirala RK, Green SR, Quehenberger O 1998 Chemokine receptor CCR2 expression and monocyte chemoattractant protein-1-mediated chemotaxis in human monocytes. A regulatory role for plasma LDL. Arteriosclerosie, Thrombosis, and Vascular Biology, 18(12): 1983–1991. 10.1161/01.atv.18.12.1983 9848893

[ZoolRes-40-4-317-R11] Hansson GK, Libby P 2006 The immune response in atherosclerosis: a double-edged sword. Nature Reviews Immunology, 6(7): 508–519. 10.1038/nri1882 16778830

[ZoolRes-40-4-317-R12] Hwang HJ, Jung TW, Kim BH, Hong HC, Seo JA, Kim SG, Kim NH, Choi KM, Choi DS, Baik SH, Yoo HJ 2015 A dipeptidyl peptidase-IV inhibitor improves hepatic steatosis and insulin resistance by AMPK-dependent and JNK-dependent inhibition of LECT2 expression. Biochemical Pharmacoloyg, 98(1): 157–166. 10.1016/j.bcp.2015.08.098 26297911

[ZoolRes-40-4-317-R13] Johnson JL 2007 Matrix metalloproteinases: influence on smooth muscle cells and atherosclerotic plaque stability. Expert Review Cardiovascular Therapy, 5(2): 265–282. 17338671 10.1586/14779072.5.2.265

[ZoolRes-40-4-317-R14] Jung TW, Chung YH, Kim HC, Abd El-Aty AM, Jeong JH 2018 LECT2 promotes inflammation and insulin resistance in adipocytes via P38 pathways. Journal of Molecular Endocrinology, 61(1): 37–45. 29650721 10.1530/JME-17-0267

[ZoolRes-40-4-317-R15] L'Hermitte A, Pham S, Cadoux M, Couchy G, Caruso S, Anson M, Crain-Denoyelle AM, Celton-Morizur S, Yamagoe S, Zucman-Rossi J, Desdouets C, Couty JP 2019 Lect2 controls inflammatory monocytes to constrain the growth and progression of hepatocellular carcinoma. Hepatology, 69(1): 160–178. 30070727 10.1002/hep.30140

[ZoolRes-40-4-317-R16] Lim S, Park S 2014 Role of vascular smooth muscle cell in the inflammation of atherosclerosis. BMB Reports, 47(1): 1–7. 24388105 10.5483/BMBRep.2014.47.1.285 PMC4163848

[ZoolRes-40-4-317-R17] Lu XJ, Chen J, Yu CH, Shi YH, He YQ, Zhang RC, Huang ZA, Lv JN, Zhang S, Xu L 2013 LECT2 protects mice against bacterial sepsis by activating macrophages via the CD209a receptor. Journal of Experimental Medicine, 210(1): 5–13. 23254286 10.1084/jem.20121466 PMC3549712

[ZoolRes-40-4-317-R18] Lu XJ, Chen Q, Rong YJ, Yang GJ, Li CH, Xu NY, Yu CH, Wang HY, Zhang S, Shi YH, Chen J 2016 LECT2 drives haematopoietic stem cell expansion and mobilization via regulating the macrophages and osteolineage cells. Nature Communications, 7: 12719. 10.1038/ncomms12719 PMC5025878 27596364

[ZoolRes-40-4-317-R19] Moore KJ, Tabas I 2011 Macrophages in the pathogenesis of atherosclerosis. Cell, 145(3): 341–355. 21529710 10.1016/j.cell.2011.04.005 PMC3111065

[ZoolRes-40-4-317-R20] Mozaffarian D, Benjamin EJ, Go AS, Arnett DK, Blaha MJ, Cushman M, Das SR, de Ferranti S, Despres JP, Fullerton HJ, Howard VJ, Huffman MD, Isasi CR, Jimenez MC, Judd SE, Kissela BM, Lichtman JH, Lisabeth LD, Liu S, Mackey RH, Magid DJ, McGuire DK, Mohler ER, 3rd, Moy CS, Muntner P, Mussolino ME, Nasir K, Neumar RW, Nichol G, Palaniappan L, Pandey DK, Reeves MJ, Rodriguez CJ, Rosamond W, Sorlie PD, Stein J, Towfighi A, Turan TN, Virani SS, Woo D, Yeh RW, Turner MB 2016 Heart disease and stroke statistics-2016 update: A report from the American Heart Association. Circulation, 133(4): e38–360. 26673558 10.1161/CIR.0000000000000350

[ZoolRes-40-4-317-R21] Müller A, Krämer SD, Meletta R, Beck K, Selivanova SV, Rancic Z, Kaufmann PA, Vos B, Meding J, Stellfeld T, Heinrich TK, Bauser M, Hütter J, Dinkelborg LM, Schibli R, Ametamey SM 2014 Gene expression levels of matrix metalloproteinases in human atherosclerotic plaques and evaluation of radiolabeled inhibitors as imaging agents for plaque vulnerability. Nuclear Medicine and Biology, 41(7): 562–569. 24853402 10.1016/j.nucmedbio.2014.04.085

[ZoolRes-40-4-317-R22] Okumura A, Saito T, Otani I, Kojima K, Yamada Y, Ishida-Okawara A, Nakazato K, Asano M, Kanayama K, Iwakura Y, Suzuki K, Yamagoe S 2008 Suppressive role of leukocyte cell-derived chemotaxin 2 in mouse anti-type II collagen antibody-induced arthritis. Arthritis & Rheumatism, 58(2): 413–421. 18240267 10.1002/art.23215

[ZoolRes-40-4-317-R23] Ovejero C, Cavard C, Périanin A, Hakvoort T, Vermeulen J, Godard C, Fabre M, Chafey P, Suzuki K, Romagnolo B, Yamagoe S, Perret C 2004 Identification of the leukocyte cell-derived chemotaxin 2 as a direct target gene of β-catenin in the liver. Hepatology, 40(1): 167–176. 15239100 10.1002/hep.20286

[ZoolRes-40-4-317-R24] Sargeant JA, Aithal GP, Takamura T, Misu H, Takayama H, Douglas JA, Turner MC, Stensel DJ, Nimmo MA, Webb DR, Yates T, King JA 2018 The influence of adiposity and acute exercise on circulating hepatokines in normal-weight and overweight/obese men. Applied Physiology, Nutrition, and Metabolism, 43(5): 482–490. 10.1139/apnm-2017-0639 29220580

[ZoolRes-40-4-317-R25] Slowik V, Apte U 2017 Leukocyte Cell-Derived Chemotaxin-2: It's role in pathophysiology and future in clinical medicine. Clinical and Translational Science, 10(4): 249–259. 28466965 10.1111/cts.12469 PMC5504477

[ZoolRes-40-4-317-R26] Wang Y, Ding WX, Li T 2018 Cholesterol and bile acid-mediated regulation of autophagy in fatty liver diseases and atherosclerosis. Biochimica et Biophysica Acta. Molecular and Cell Biology of Lipids, 1863(7): 726–733. 29653253 10.1016/j.bbalip.2018.04.005 PMC6037329

[ZoolRes-40-4-317-R27] Wolf D, Ley K 2019 Immunity and inflammation in atherosclerosis. Circulation Research, 124(2): 315–327. 30653442 10.1161/CIRCRESAHA.118.313591 PMC6342482

[ZoolRes-40-4-317-R28] Yamagoe S, Mizuno S, Suzuki K 1998 Molecular cloning of human and bovine LECT2 having a neutrophil chemotactic activity and its specific expression in the liver. Biochimica et Biophysica Acta, 1396(1): 105–113. 9524238 10.1016/s0167-4781(97)00181-4

[ZoolRes-40-4-317-R29] Ye J, DeBose-Boyd RA 2011 Regulation of cholesterol and fatty acid synthesis. Cold Spring Harbor Perspectives in Biology, 3(7): a004754. 21504873 10.1101/cshperspect.a004754 PMC3119913

[ZoolRes-40-4-317-R30] Yoo HJ, Choi KM 2015 Hepatokines as a link between obesity and cardiovascular diseases. Diabetes & Metabolism Journal, 39(1): 10–15. 25729707 10.4093/dmj.2015.39.1.10 PMC4342531

[ZoolRes-40-4-317-R31] Yoo HJ, Hwang SY, Choi JH, Lee HJ, Chung HS, Seo JA, Kim SG, Kim NH, Baik SH, Choi DS, Choi KM 2017 Association of leukocyte cell-derived chemotaxin 2 (LECT2) with NAFLD, metabolic syndrome, and atherosclerosis. PLoS One, 12(4): e0174717. 28376109 10.1371/journal.pone.0174717 PMC5380318

[ZoolRes-40-4-317-R32] Zhang Z, Zeng H, Lin J, Hu Y, Yang R, Sun J, Chen R, Chen H 2018 Circulating LECT2 levels in newly diagnosed type 2 diabetes mellitus and their association with metabolic parameters: An observational study. Medicine, 97(15): e0354. 29642178 10.1097/MD.0000000000010354 PMC5908589

[ZoolRes-40-4-317-R33] Zhong S, Zhao L, Li Q, Yang P, Varghese Z, Moorhead JF, Chen Y, Ruan XZ 2015 Inflammatory stress exacerbated mesangial foam cell formation and renal injury via disrupting cellular cholesterol homeostasis. Inflammation, 38(3): 959–971. 25387652 10.1007/s10753-014-0058-0

